# Extraction and purification of high-value metabolites from microalgae: essential lipids, astaxanthin and phycobiliproteins

**DOI:** 10.1111/1751-7915.12167

**Published:** 2014-09-15

**Authors:** Sara P Cuellar-Bermudez, Iris Aguilar-Hernandez, Diana L Cardenas-Chavez, Nancy Ornelas-Soto, Miguel A Romero-Ogawa, Roberto Parra-Saldivar

**Affiliations:** Cátedra de Bioprocesos Ambientales, Centro del Agua Para América Latina y el Caribe, Instituto Tecnológico y de Estudios Superiores de MonterreyMonterrey, Nuevo Leon, 64849, Mexico

## Abstract

The marked trend and consumers growing interest in natural and healthy products have forced researches and industry to develop novel products with functional ingredients. Microalgae have been recognized as source of functional ingredients with positive health effects since these microorganisms produce polyunsaturated fatty acids, polysaccharides, natural pigments, essential minerals, vitamins, enzymes and bioactive peptides. For this reason, the manuscript reviews two of the main high-value metabolites which can be obtained from microalgae: pigments and essential lipids. Therefore, the extraction and purification methods for polyunsaturated fatty acids, astaxanthin, phycoerythrin and phycocyanin are described. Also, the effect that environmental growth conditions have in the production of these metabolites is described. This review summarizes the existing methods to extract and purify such metabolites in order to develop a feasible and sustainable algae industry.

## Introduction

Microalgae are eucaryotic photosynthethic microorganisms that use solar energy, nutrients and carbon dioxide (CO_2_) to produce proteins, carbohydrates, lipids and other valuable organic compounds like carotenoids (Mendes *et al*., 2003; Batista *et al*., 2013). The chemical compounds synthesized by microalgae have several applications. The high-protein content of some algae species is one of the main reasons to consider them as a non-conventional source of proteins. For example, *Chlorella* and *Spirulina* were the first microalgae species to be commercialized as a health food in Japan, Taiwan and Mexico (Sánchez *et al*., 2003; Borowitzka, 2013). However, information on the nutritional value of the protein and the degree of availability of the amino acids should be stated (Spolaore *et al*., 2006). Carbohydrates like starch, glucose, sugars and other polysaccharides have high overall digestibility for food or feeds (Spolaore *et al*., 2006). As an example, macroalgal polysaccharides like agar, alginates and carrageenans are used in diverse fields of industry due to their rheological gelling or thickening properties (Pulz and Gross, 2004). Finally, lipids and fatty acids from microalgae including the omega-3 (ω3) and omega-6 (ω6) families, have gained particular interest due to the health benefits related to its consumption (Spolaore *et al*., 2006). Aquaculture is also one of the main applications of lipids and pigments produced by algae since these microorganisms are essential in the food chain. Therefore, different studies have been carried in order to identify potential algae species to be used in aquaculture (Gladue and Maxey, 1994; Borowitzka, 1997; Makri *et al*., 2011; Birkou *et al*., 2012).

Depending on the microalgae species, various high-value compounds can be extracted from the biomass (Table 1). These include pigments, polysaccharides, triaglycerides, fatty acids and vitamins, which are also commonly used as bulk commodities and specialty chemicals in different industrial sectors (e.g. pharmaceuticals, cosmetics, nutraceuticals, functional foods, aquaculture, biofuels). High-value products from microalgae are usually produced within a biorefinery model, since the composition of the microalgal cell allows for extraction of different co-products. In addition, specialty chemicals have higher revenues than bulk chemicals like algal oil for biofuels (Sharma *et al*., 2010; Borowitzka, 2013; Gerardo *et al*., 2014). Different commercialization prices of algae products have been reported (Table 2). Some reports show a decrease in the marketing prices (except for omega-3). This can be explained by the fact that more companies are focusing in the production and commercialization of compounds from microalgae (Table 3). Nevertheless, prices are still high due to the process expenses associated to the extraction and purification of intracellular metabolites. In microalgal biotechnological processes, the downstream stage can account for 50–80% of total production costs, depending on the biochemical characteristics of the compound and the purity ratio that needs to be achieved. (Molina Grima *et al*., 2003).

**Table 1:** Microalgae species of high-value compounds extraction and applications (Pulz and Gross, 2004; Spolaore *et al*., 2006; Casal *et al*., 2011; Guedes *et al*., 2011; Batista *et al*., 2013; Borowitzka, 2013; Sørensen *et al*., 2013)

Species	Product	Application areas
*Chlorella vulgaris*	Biomass, pigments	Health food, food supplement
*Chlorella* spp.		
*Chlorella ellipsodea*		
*Coccomyxa acidophila*	Lutein, β-carotene	Pharmaceuticals, nutrition
*Coelastrella striolata var. multistriata*	Canthaxanthin, astaxanthin, β-carotene	Pharmaceuticals, nutrition, cosmetics
*Crypthecodinium conhi*	Docosahexaenoic acid	Pharmaceuticals, nutrition
*Diacronema vlkianum*	Fatty acids	Pharmaceuticals, nutrition
*Dunaliella salina*	Carotenoids, β-carotene	Health food, food supplement, feed
*Galdiera suphuraria*	Phycocyanin	Pharmaceuticals, nutrition
*Haematococcus pluvialis*	Carotenoids, astaxanthin, cantaxanthin, lutein	Health food, pharmaceuticals, feed additives
*Isochrysis galbana*	Fatty acids, carotenoids, fucoxanthin	Pharmaceuticals, nutrition, cosmetics, animal nutrition
*Lyngbya majuscule*	Immune modulators	Pharmaceuticals, nutrition
*Muriellopsis sp.*	Lutein	Pharmaceuticals, nutrition
*Nannochloropsis gaditana*	Eicosapentaenoic acid	Pharmaceuticals, nutrition
*Nannochloropsis* sp.		
*Odontella aurita*	Fatty acids	Pharmaceuticals, cosmetics, baby food
*Parietochloris incise*	Arachidonic acid	Nutritional supplement
*Phaedactylum tricornutum*	Lipids, eicosapentaenoic acid, fatty acids	Nutrition, fuel production
*Porphyridium cruentum*	Arachidonic acid, polysaccharides	Pharmaceuticals, cosmetics, nutrition
*Scenedesmus almeriensis*	Lutein, β-carotene	Pharmaceuticals, nutrition, cosmetics
*Schizochytrium* sp.	Docosahexaenoic acid	Pharmaceuticals, nutrition
*Spirulina platensis*	Phycocyanin, γ-Linolenic acid, biomass protein	Health food, cosmetics
*Ulkenia* spp.	Docosahexaenoic acid	Pharmaceuticals, nutrition

**Table 2:** Commercialization price of some high value compounds from algae

Product	Price (€) Spolaore *et al*., 2006a	Price (€) Brennan and Owende, 2010	Price (€) Markou and Nerantzis, 2013a	Price (€) Borowitzka, 2013a
Phycobiliproteins	—	11–50 mg^−1^	—	
B-phycoerythrin	105 mg^−1^	—	0.036 mg^−1^	
C-phycocyanin				360–72 460 Kg^−1^
β-Carotene	—	215–2150 Kg^−1^	218–510 Kg^−1^	
Astaxanthin	—	7150 Kg^−1^	1,450–5075 Kg^−1^	
DHA oil/Omega-3	—	0.043 Kg^−1^	0.63–2.78 Kg^−1^	78–116 Kg^−1^

a Price expressed in USD by the authors. Conversion factor of 1.38.

**Table 3:** Global companies based in developing process and commercialization of high-value compounds from algae

Company name	Location	Company name	Location
Algae. Tec	Australia	AlgaFuel, S.A (A4F)	Portugal
Solarvest BioEnergy	Canada	Necton	Portugal
Canadian Pacific Algae	Canada	Green Sea Bio Systems s.l.	Spain
Solarium Biotechnology S.A.	Chile	AlgaEnergy	Spain
BlueBio	China	Fitoplancton Marino	Spain
EcoFuel Laboratories	Czech Republic	Simris	Sweden
Aleor	France	Taiwan Chlorella Manufacturing Company	Taiwan
Fermentalg	France	Vedan	Taiwan
Roquette	France	AlgaeLink N.V	The Netherlands
Alpa Biotech	France	AlgaeBiotech	The Netherlands
IBV Biotech IGV GmbH	Germany	LGem	The Netherlands
Subitec	Germany	Solazyme, Inc.	USA
Algomed	Germany	Aurora Algae	USA
BlueBioTech	Germany	Solix Biosystems	USA
Phytolutions	Germany	Synthetic Genomics	USA
Algae Health	Ireland	Cellena	USA
Seambiotic	Israel	Cyanotech	USA
Algatechnologies	Israel	Algaeon	USA
UniVerve Biofuel	Israel	Alltech Algae	USA
Parry Nutraceuticals	India	Green Star Products, Inc	USA
Sunchlorella	Japan	Bionavitas	USA
Fuji Chemicals	Japan	Heliae	USA
DAESANG	Korea	Kuehnle Agro Systems	USA
Algaetech International	Malaysia	Photon8	USA
June Pharmaceutical	Malaysia	Ternion BioIndustries	USA
Tecnología Ambiental BIOMEX	Mexico	Algae to Omega Holdings	USA
Algae Technology Solutions	Mexico	Sapphire Energy	USA
Aquaflow Binomics	New Zealand	Algenol	USA
Photonz	New Zealand		

This article, based on literature framework, describes lipids and pigments as two representative classes of high-value compounds synthesized by algae. In the case of lipids, metabolic production, extraction and quantification methods are discussed. For pigments, the extraction and purification methods for astaxanthin, phycocyanin and phycoerytrin are described.

## Lipids and polyunsaturated fatty acids

The main components of the algae lipid fraction are fatty acids (FA), waxes, sterols, hydrocarbons, ketones and pigments (carotenoids, chlorophylls, phycobilins) (Halim *et al*., 2011). Depending on the species, total lipids in microalgae usually represent 20% to 50% of total biomass in dry weight (DW). However, other values have also been reported in a range from 1% to 70% (Spolaore *et al*., 2006). Fatty acids can generally be classified into two categories based on the polarity of the molecular head group: (i) neutral lipids which comprise acylglycerols and free fatty acids (FFA) and (ii) polar lipids or amphipathic lipids which can be further subcategorized into phospholipids and glycolipids. Acylglycerols consist of fatty acids ester bonded to a glycerol backbone, and according to its number of fatty acids are categorized in triacylglycerides (TAG), diacylglycerols, monoacylglycerols (Halim *et al*., 2011).

Lipid production by microalgae depends on the species and is affected by culture conditions such as nutrients, salinity, light intensity periods, temperature, pH and even the association with other microorganisms (Richmond, 2004; Guschina and Harwood, 2006). Nitrogen limitation is considered the most efficient strategy to increase the content of neutral lipids in algae, mainly formed by the triglycerides with high degree of saturation. However, a decrease in biomass productivity occurs. Breuer and colleagues (2012) reported an increase in the TAG accumulation as response of nitrogen limitation in *Chlorella vulgaris, Chlorella zofingiensis, Neochloris oleoabundans* and *Scenedesmus obliquus.* Total lipid content in *Ulva pertusa*, *Euglena gracilis* and *Botryococcus* species increased with nitrogen starvation (Floreto *et al*., 1993; Regnault *et al*., 1995; Yeesang and Cheirsilp, 2011). In addition, in contrast to the polar lipids of nitrogen-sufficient cells, neutral lipids in the form of triacylglycerols become predominant in lipids from nitrogen-depleted cells (Richmond, 2004).

As mentioned, nitrogen starvation is one the main strategies to increase lipid and TAG production by algae. However, it has also been reported that nitrogen starvation favours biosynthesis of starch (Ramazanov and Ramazanov, 2006; Wang *et al*., 2009). Phosphorous limitation causes the replacement of membrane phospholipids by non-phosphorus glycolipids representing and effective phosphate-conserving mechanism. However, Guschina and colleagues (2003) reported that algae under phosphorous limitation, maintain their phosphoglyceride synthesis since significant endogenous phosphorus stores in the algae were found by X-ray electron microscopy. In case of the light intensity effect, diverse studies suggest that high light intensity and therefore high temperature favour the accumulation of triglycerides with high saturation profile (Floreto *et al*., 1993; Van Wagenen *et al*., 1999). Meanwhile, low light intensities and low temperature promote the synthesis of polyunsaturated fatty acids (PUFA) (Guschina and Harwood, 2006). The effect of carbon dioxide concentration has been studied in *C. Kessleri*, low-CO_2_ cultures showed high contents of α-linolenate fatty acid (Sato *et al*., 2003). In contrast, in *C. reinhardtii* mutant cia-3, higher content of PUFA was found in cultures with high CO_2_ concentration. Finally, pH can also affect the lipid metabolism. Low pH stress in *Chlamydomonas* sp. increased the total lipid content compared with higher pH values (Tatsuzawa *et al*., 1996). However in *Chlorella* spp., alkaline pH resulted in triacylglycerides accumulation (Guckert and Cooksey, 1990).

Fatty acids biosynthesis carries out by the conversion of acetyl coenzyme A (acetyl-CoA) to malonyl-CoA, catalysed by the complex enzyme acetyl-CoA carboxylase. One of the main biochemical pathways for acetyl-CoA production comes from the 3-phosphoglycerate (3-PG), primary product of carbon dioxide fixation. Also, 3-PG is also the precursor for the glycerol backbone of TAG, however, 3-PG also participates into the starch biosynthesis pathway (Wang *et al*., 2009). Therefore, studies have been carried in order to identify the biochemical pathways for polysaccharides and lipids production in algae (Bellou and Aggelis, 2012). Also, to increase lipid and TAG accumulation in cells, non-starch algae mutant strains are been studied and characterized (Ramazanov and Ramazanov, 2006; Wang *et al*., 2009).

Omega-3 (ω-3) PUFA are a specific group of polyunsaturated fatty acids in which the first double bond is located between the third and fourth carbon atom counting from the methyl end of the fatty acid (Ryckebosch *et al*., 2012). Fish oil is a major non-sustainable source for the commercial production of these fatty acids. Fish oil quality depends on the fish species, the season/climate and geographical location of catching sites and food quality consumed by the fish. In addition, fish oil is not suitable for vegetarians, and its odour makes it unattractive for consumption. Moreover, in some cases, there is a contamination danger by lipid-soluble environmental pollutants (Ryckebosch *et al*., 2012).

In addition, PUFAs are subject of intensive research due to the important health benefits associated with their consumption (Wen and Chen, 2003; Sijtsma and de Swaaf, 2004). These include the α-Linolenic acid (ALA 18:3 ω-3), γ-Linolenic acid (GLA, 18:3 ω-6), eicosapentaenoic acid (EPA, 20:5 ω-3), arachidonic acid (ARA, 20:6 ω-6) docosapentaenoic acid (22:5 ω-3) and docosahexaenoic acid (DHA, 22:6 ω-3) (Fraeye *et al*., 2012; Ryckebosch *et al*., 2012). According to Adarme-Vega and colleagues (2012), these long chain ω-3 PUFA provide significant health benefits particularly in reducing cardiac diseases such as arrhythmia, stroke and high blood pressure. As well, they have beneficial effects against depression, rheumatoid arthritis, asthma and can be used for treatment of inflammatory diseases such as rheumatoid arthritis, Crohn's disease, ulcerative colitis, psoriasis, lupus and cystic fibrosis. Additionally, in pregnant women, the adequate intake of EPA and DHA is crucial for healthy development of the fetal brain. In addition, ARA and DHA are required for normal growth and brain functional development, while EPA is essential for the regulation of some biological functions and prevention of arrhythmia, atherosclerosis, cardiovascular disease and cancer (Pulz and Gross, 2004). In Table 4 are shown some microalgae species source of high-quality PUFAs. In addition, the extraction method and yield of the FA is listed.

**Table 4:** PUFAs extracted in different microalgae species

Species	Product	Yield	Extraction/Purification method	Reference
*Arthrospira platensis*	Total lipids	13.2% DW	Chloroform–methanol 1:1 (%v/v)	Ryckebosch and colleagues (2011)
*Chlorella vulgaris*		19.9% DW		
*Chlorella vulgaris* Green cells	ALA	661 mg/100 g	Acid digestion of biomass with 4 N HCl.	Batista and colleagues (2013)
	EPA	19 mg/100 g		
	DHA	16 mg/100 g	Soxhlet method with petroleum ether for 6 h	
	GLA	112 mg/100 g		
*Chlorella vulgaris* Orange cells	ALA	3665 mg/100 g		
	EPA	39 mg/100 g		
	DHA	80 mg/100 g		
	GLA	23 mg/100 g		
*Crypthecodinium cohnii*	DHA	99.2% purity	Purification by saponification, winterization and urea complexation	Mendes and colleagues (2007)
*Diacronema vlkianum*	ALA	14 mg/100 g	Acid digestion of biomass with 4 N HCl.	Batista and colleagues (2013)
	EPA	3212 mg/100 g		
	DHA	836 mg/100 g	Soxhlet method with petroleum ether for 6 h	
	GLA	112 mg/100 g		
*H. pluvialis*	ALA	3981 mg/100 g		
	EPA	579 mg/100 g		
	GLA	472 mg/100 g		
*Isochrysis galbana*	ALA	421 mg/100 g		
	EPA	4875 mg/100 g		
	DHA	1156 mg/100 g		
*Scenedesmus obliquus*	Total Lipids	29.7% DW	Chloroform–methanol 1:1 (%v/v)	Ryckebosch and colleagues (2011)
*Spirulina maxima*	ALA	40 mg/100 g	Acid digestion of biomass with 4 N HCl. Soxhlet method with petroleum ether for 6 h	Batista and colleagues (2013)
	GLA	452 mg/100 g		
*Nannochloropsis salina*	Total Lipids	34.4% DW	Chloroform–methanol 1:1 (%v/v)	Ryckebosch and colleagues (2011)
*Nannochloropsis gaditana*	EPA	3.7 g/100 g DW	Dichloromethane-ethanol (1:1)	Ryckebosch and colleagues (2013)
*Parietochloris incise*	ARA	9.1% DW	Methanol (10%DMSO) at 40°C for 5 min.	Bigogno and colleagues (2002)
			Diethyl ether, hexane and water 1:1:1 (v/v/v)	
*Phaeodactylum tricornutum*	EPA	9.3% DW of total fatty acids (39%)	Chloroform–methanol 1:1 (%v/v)	Ryckebosch and colleagues (2011)
*Porphyridium cruentum*	EPA	50.8% recovery, 97% purity	Purification by saponification and urea complexation	Guil-Guerrero and colleagues (2000)
*Tetraselmis sp.*	EPA	10.41% of phospholipid fraction	Chloroform-methanol 2:1 (%v/v). Extract was washed with 0.88% w/v KCl to remove non-lipids	Makri and colleagues (2011)

**Figure 1:**
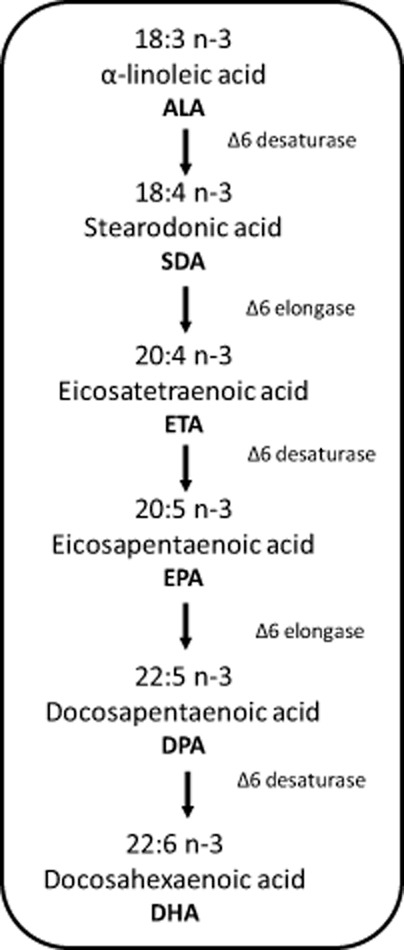
Pathway for the biosynthesis of omega-3 LC-PUFA (Wen and Chen, 2003; Adarme-Vega *et al*., 2012; Ryckebosch *et al*., 2012).

The biosynthesis of EPA/DHA (Fig. 1) occurs through a series of reactions which can be divided in two different steps. First is the novo synthesis of oleic acid (18:1) from acetate. This is followed by conversion of oleic acid (18:1) to linoleic acid (LA, 18:2) and α-linolenic acid (18:3). Finally, after a number of subsequent stepwise desaturation and elongation steps, the ω-3 PUFA family including EPA and DHA are formed (Wen and Chen, 2003).

In microalgae, EPA is found in the *Bacillariophceae* (diatoms) *Chlorophyceae, Chrysophyseae, Cryptophyceae, Eustigamatophyceae* and *Prasinophyceae* classes (Singh *et al*., 2005). The heterotrophic marine dinoflagellate *Crypthecodinium cohnii*, together with *Schizochytrium* and related genera, represent the major commercial and interesting source of DHA. *Crypthecodinium cohnii* can accumulate a high percentage of DHA (25–60% of the total fatty acids) as TAG with only trivial amounts of other fatty acids (Mendes *et al*., 2007; Zvi and Colin, 2010). In addition, other algae species have been studied as source of PUFA. Chen and colleagues (2007) analysed the microalgae *Nitzschia laevis*, founding that 75.9% of total EPA was accumulated as 37.4% in TAG, 22.6% in monoacylglycerides and 15.9% in phosphatidylcholine. Bigogno and colleagues (2002) analysed fatty acid production by the green algae *Parietochloris incise* concluding that ARA comprised 33.6% of total fatty acids in the logarithmic phase and 42.5% in the stationary phase with a 27% of total fatty acids.

### Extraction and quantification methods

Historically, the three most common processes for recovering oil from most plant seeds are hydraulic pressing, expeller pressing and solvent extraction. Solvent extraction was originated as a batch process in Europe in 1870 (Zvi and Colin, 2010). Currently, algae oil extraction procedures include mechanical pressing, homogenization, milling and solvent extraction. The most common solvents for lipid extraction are chloroform–methanol, hexane, hexane–isopropanol or other solvent mixtures slightly soluble in each other. Depending on polarity and/or solubility of the lipid content, the adequate solvent or mixture must be chosen for the extraction.

Ryckebosch and colleagues (2011) tested different solvents for lipid extraction in four algae species *(Nannochloropsis salina, S. obliquus, C. vulgaris and A Arthrospira platensis)* finding that chloroform–methanol 1:1 (%v/v) allows the highest lipid extraction and is thus the preferred solvent mixture for total lipids determination. Moreover, while this analysis is performed, lyophilized algae can be used without the need for biomass pretreatment. This refers to the addition of isopropanol or antioxidants to inactivate the lipases for further oxidation. Also, the authors concluded that no cell disruption method is necessary, and in general two series of solvent extractions must be carried out. However, other methods such as microwaves, supercritical fluid extraction, enzymatic extractions, ultrasonic-assisted extraction, pulsed electric field technology and osmotic shock are used to enhance the extraction of cellular lipids bodies by solvents (Mercer and Armenta, 2011; Zheng *et al*., 2011; Goettel *et al*., 2012).

Lipids also need to be separated from carbohydrates, proteins or salts to enhance extraction or analysis. Acid hydrolysis has been used to break the lipids bonds with other compounds, increasing the extraction yield. As an example, hydrochloric acid or glacial acetic acid are used as pretreatment or mixed with the solvent used in the Soxhlet extraction (Palmquist and Jenkins, 2003). The moisture content in a sample can affect the performance of the lipid extraction (Griffiths *et al*., 2010).

Goettel and colleagues (2012) studied the effect of pulsed electric field treatment as a cell disintegration method altering the structure of the cell membrane (permeability) in *Auxenochlorella protothecoides*. The results showed an increment in conductivity and a drop of pH of the treated suspensions due to the release of soluble cell material; however the lipid droplets remained inside the cells. Therefore, the authors proposed a two-stage process, first for water soluble cell materials extraction and subsequently for material soluble in organic solvents.

Most authors reporting lipid determination commonly use chromatography with flame-ionization detectors after transesterification of fatty acids (acylglycerols) with alcohol (methanol) to produce fatty acid methyl esters. However, some other techniques are currently used: fluorometry, colourimetry, Raman spectroscopy, gas chromatography with mass spectrometric detector, high performance liquid chromatography (HPLC) with pulsed amperometric detection, reverse phase HPLC, UV detection at 205 nm, evaporative light scattering detection, atmospheric pressure chemical ionization mass spectrometry, nuclear magnetic resonance, near infrared and Fourier transform infrared spectroscopy (Cooksey *et al*., 1987; Lee *et al*., 1998; Knothe, 2001; Meher *et al*., 2006; Elsey *et al*., 2007; Huang *et al*., 2010; Laurens and Wolfrum, 2010; Wawrik and Harriman, 2010; Cheng *et al*., 2011; Davey *et al*., 2012; De la Hoz Siegler *et al*., 2012).

Diverse technologies besides solvent extraction have been developed to achieve fast quantifications of the lipid content in algae cultures in order to determine the optimal time for harvesting (De la Hoz Siegler *et al*., 2012). These techniques are non-destructive, and just small samples volume is needed (Davey *et al*., 2012). Specifically, fluorescence methods are used for these purposes. However, fluorescence intensity is affected by several factors as staining agent concentration, cell concentration, staining temperature and staining duration (De la Hoz Siegler *et al*., 2012). Therefore, according to the algae species, the stain method should be optimized. De la Hoz Siegler and colleagues (2012) studied the effects that biomass and staining concentration, and mixing time have on the results variability of Nile Red (9-diethylamino-5H-benzo[α]-phenoxazine-5-one) fluorescence method for lipid determination in 4 algae species: *C. vulgaris, A. protothecoides, Scenedesmus dimorphus* and *S. obliquus.* As well, the addition of ethanol as carrier solvent was also evaluated. The results showed that staining time less than 20 min did not affect the measurement error, and 10 μg/ml of Nile Red solution was an appropriate concentration for each 10 μg/ml of algal suspension (5 g/L DW). Moreover, the sample standard deviation was reduced by 56% when all the reagents were mixed before distributing the sample into the microwells. In comparison, Chen and colleagues (2011) used microwaves in combination with dimethyl sulfoxide (DMSO) for an effective staining of lipid bodies. However, DMSO could carry other compounds increasing the error measurement (De la Hoz Siegler *et al*., 2012).

### Purification methods

Docosahexaenoic acid/eicosapentaenoic acid crude oil is unfit for consumption because of its impurities, odour and taste, as well as cloudy or turbid appearance. Hence, it needs to be refined. This process can be achieved applying standard vegetable oil refining steps as degumming, caustic refining, bleaching and deodorization. However, as the oil is sensitive to oxidation, process conditions and speed of operation are critical. Specifically, Martek Biosciences Corp. has developed an optimized method of purification conditions based in parameters including odour, taste and oxidative stability. In addition, the deodorized oil is blended with high oleic antioxidants, mainly, ascorbyl palmitate and tocopherols (Martek Biosciences Corporation, 2010; Zvi and Colin, 2010).

Guil-Guerrero and colleagues (2000) purified EPA from *Porphyridium cruentum* by simultaneous oil saponification (potassium hydroxide and ethanol at 60°C for 1 h) and fatty acid extraction of the microalgal biomass. After this, PUFA were concentrated by the urea method (methanol/urea ratio 3:1 w/w, crystallization temperature 28°C) and the concentrate was transmethylated (with acetyl chloride and methanol). Finally, EPA methyl esters were separated from PUFA concentrate by an argentated silica gel column chromatography with recoveries of 50.8% of EPA with 97% of purity.

Mendes and colleagues (2007) developed a procedure to concentrate DHA from *C. cohnii* involving saponification and methylation in wet biomass for further winterization and urea complexation. The process yield was 99.2% of total fatty acids. The urea/fatty acid ratio used was 3.5, and the temperatures of crystallization were 4°C and 8°C. Winterization (− 18°C) was used to fraction TAG with different melting points that are present in edible oils allowing the solid portion to crystallize. Subsequently, filtration of the two phases was performed. At low temperatures, long-chain saturated fatty acids, which have higher melting points, crystallize out and the PUFA remain in the liquid form, while urea molecules readily form solid-phase complexes with saturated FFA. In addition, urea complexation allows the handling of large quantities of material in simple equipment, requiring inexpensive solvents such as methanol or hexane. Finally, the separation is more efficient than with other methods such as fractional crystallization or selective solvent extraction. Besides reducing costs, urea complexation protects the ω-3 PUFA from autoxidation.

Oil recovery and purification process of DHA from algae oil has also been described in the petition of Market Biosciences Corporation (Martek Biosciences Corporation, 2010). For *Schizochytrium sp*., a protease enzyme breaks the proteins in the cell releasing the oil from the cells into the culture broth forming an oil and water emulsion. Isopropyl alcohol is then added to break the emulsion and separate the oil. Subsequently, the alcohol is recovered from the broth, evaporated and distilled for reusing in subsequently extractions. In contrast, *C. cohnii* cannot be hydrolysed by enzymes because of the cellulosic thecal layer. Consequently, hexane solvent extraction on dry biomass is required. Later, the cell walls are removed through centrifugation, and the oil is recovered after solvent evaporation. Oil purification includes oil heating followed by the addition of an acidulated solution (oleic or citric), and later sodium hydroxide is added. These pH changes cause the formation of ‘soaps’ and ‘gums’ that can be easily separated and removed. Oil is reheated and centrifuged to separate the refined oil. Later, sand chelators as citric acid, silica or clay are used to remove remaining residual compounds. Finally, the treated oil may be chill filtered to remove high-melting point components (stearines and waxes) to achieve the desired level of clarity. Subsequently, the oil is heated and then cooled to crystallize triacylglycerols and waxes. Later, diatomaceous earth is added to the chilled oil, and the crystallized solids are removed by filtration. Deodorizer is used to remove peroxides and the remaining low molecular weight compounds such as carbonyls and aldehydes that may cause off odours and flavours. Finally, tocopherols, rosemary extracts and ascorbyl palmitate are added to provide oxidative stability and flavour to the refined oil.

## Natural pigments

Among functional ingredients identified from marine algae, natural pigments (NPs) have received particular attention. Natural pigments have an important role in the photosynthetic and pigmentation metabolism of algae, and also exhibit several beneficial biological activities like antioxidant, anti-carcinogenic, anti-inflammatory, anti-obesity, anti-angiogenic and neuroprotective (Guedes *et al*., 2011; Pangestuti and Kim, 2011).

The three basic classes of NPs found in marine algae are chlorophylls, carotenoids and phycobiliproteins. Table 5 summarizes the main microalgal species used as a source of natural pigments along with the extraction and purification methods currently used for each species.

**Table 5:** Extraction of natural pigments in microalgae species

Species	Product	Extraction/Purification method	Yield/Extraction efficiency	Reference
*Chlorococcum* sp.	Astaxanthin	Solvent system with methanol (75%) and dichloromethane (25%). French Pressure Cell (110 MPa). Solution filtration (0.45 μm). Saponification in darkness (50 mg NaOH in 100 ml methanol).	7.09 mg/g DW	Ma and Chen (2001)
*Haematococcus pluvialis*	Astaxanthin	Acid digestion, HCL 2N, 70°C. Acetone extraction for 1 h	87% efficiency	Sarada and colleagues (2006)
		Dodecane mixing for 48 h. Saponification with methanolic NaOH (0.02M). Sedimentation in darkness at 4°C, 12 h.	85% efficiency	Kang and Sim (2007)
		SC-CO_2_ at 55 MPa and 343°K.	77.9% efficiency	Machmudah and colleagues (2006)
		Hexane : acetone : ethyl alcohol (100:70:70 %v/v).	N/A	Domínguez-Bocanegra and colleagues (2004)
		DMSO (55°C), vortex 30 s	N/A	Orosa and colleagues (2005)
		SC-CO_2_ at at 20 MPa, 60°C, 2 ml of ethanol for 1 h of extraction time	2.45 mg/g DW	Fujii (2012)
		SC-CO_2_ at 20 MPa, 55°C and13% (w/w) ethanol for 120 min of extraction time.	83% recovery	Reyes and colleagues (2014)
		CO_2_ expanded ethanol (50% %w/w ethanol), 7 MPa, 45°C, 120 min of extraction time.	124.2% recovery	
*Synechococcus 833*	Allophycocyanin	Incubation of sample for 2 h at 37°C, nitrogen cavitation cycles at 1500 psi for 10 min, centrifugation for 40 min at 18 000 rpm to remove cell debris.	85.2–87.9% DW	Viskari and Colyer (1999)
	C-phycocyanin			
	Phycoerythrin			
*Limnothrix* sp.	C-phycocyanin	Distilled water, activated carbon (1% w/v) and chitosan (0.01 g/L) for extraction. Ammonium sulfate (25%) was used for purification at 4°C, overnight. Precipitate was resuspended in 0.1 M PBS (pH 7.0) and tangential flow filtration system (30 kDa membrane pore) was used for pigment concentration.	18% DW	Gantar and colleagues (2012)
*Spirulina platensis*	C-phycocyanin	0.1 M PBS at pH of 6.8 and sonication at 28 KHz for extraction. Ultracentrifugation at 200 000 × g for purification	90% purity	Furuki and colleagues (2003)
	Allophycocyanin C-phycocyanin	100 mM phosphate buffer (pH 7.0) at a ratio of 1:100 (w/v) with continuous stirring at 300 rpm at room temperature for 4 h.	N/A	Chaiklahan and colleagues (2012)
*Leptolyngbya* sp. *KC45*	Phycoerythrin	Ammonium sulfate at 85% saturation. Purification by three consecutive chromatographic steps; hydroxyapatite column eluted with 100 mM phosphate buffer (pH 7) 0.2 M of NaCl, a Q-sepharose column and a Sephacryl S-200 HR resin.	A_565_/A_280_ = 17.3	Pumas and colleagues (2012)
			1.36% yield	
*Porphyridium cruentum*	Phycoerythrin	Homogenization in 1 M acetic acid–sodium acetate buffer sonication for 10 min, ammonium sulfate precipitation (65% saturation) and dialysis, followed by ion exchange chromatography.	32.7% DW	Bermejo Román and colleagues (2002)
		Cell maceration with glass beads, simultaneous recovery and purification with a PEG-phosphate ATPS.	A_565_/A_280_ = 2.8	Benavides and Rito-Palomares (2006)
			76% recovery	
*Phormidium sp*. A27DM	Phycoerythrin	Freeze-thaw cycles (− 30^o^C and 4^o^C) in 1 M Tris Cl Buffer, two-step ammonium sulfate precipitation at 20% and 70% saturation and purification by gel permeation chromatography with a Sephadex G-150 matrix.	A_565_/A_280_ = 3.9	Parmar and colleagues (2011)
			62.6% yield	

DW, dry weight.

**Figure 2:**
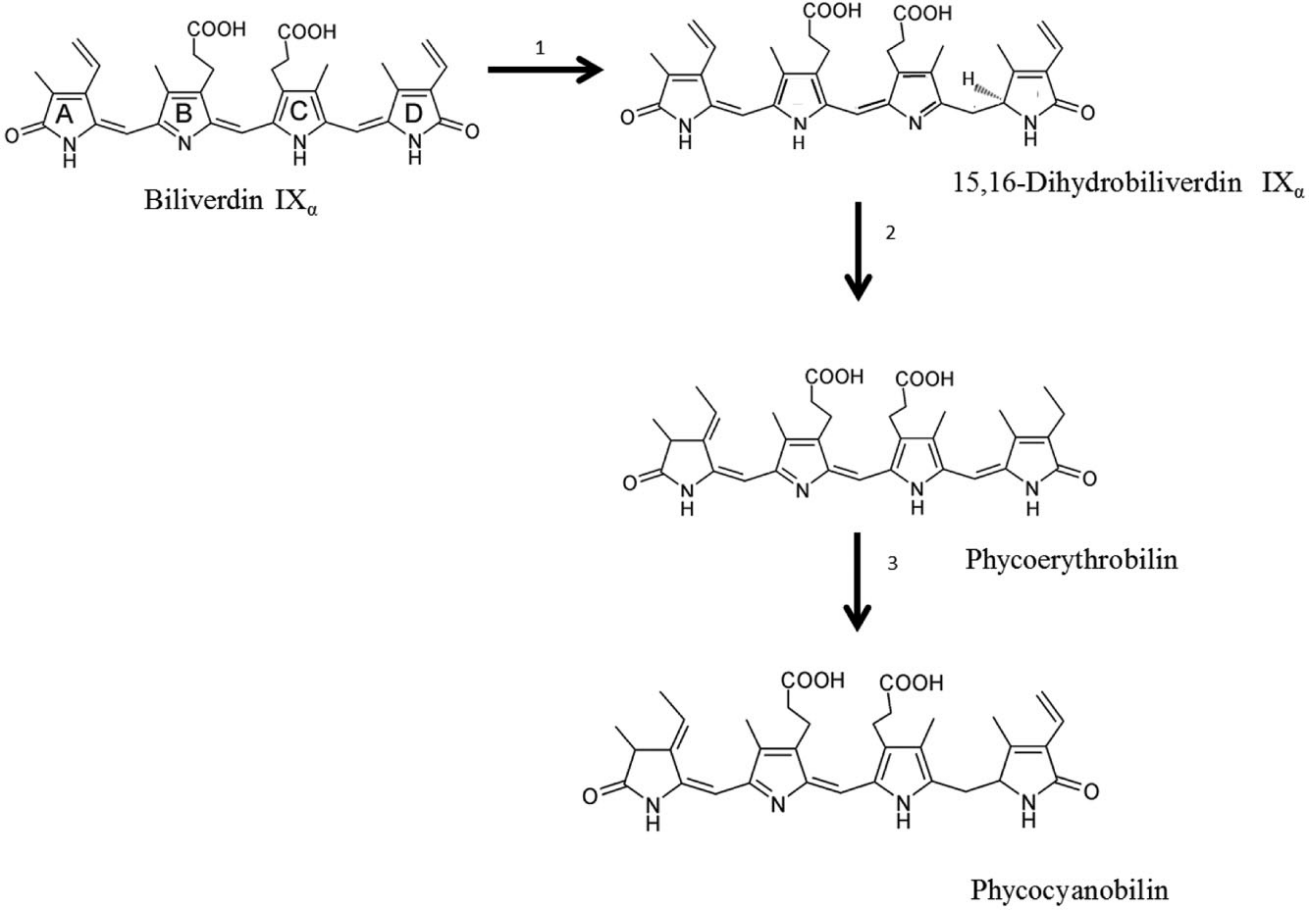
Biosynthesis pathway for phycobilins formation from Biliverdin (Brown *et al*., 1984; Lamparter *et al*., 2002). Reactions are: (i) biliverdin 15,16-reductase, (ii) bilin 2,3-reductase (iii) phycobilin (15,16-metylene-to-18^2^,18^3^-ethyl) isomerase.

Chlorophylls are greenish lipid-soluble photosynthetic pigments with a porphyrin ring in their structure. Chlorophylls are found in algae, higher plants and cyanobacteria. Carotenoids are linear polyenes that act as light energy harvesters and antioxidants that inactivate reactive oxygen species (ROS) formed by the exposure of cells to environmental stress (Ioannou and Roussis, 2009). Carotenoids are considered accessory pigments since they increase the light-harvesting properties of algae by passing the light excitation to chlorophylls (Larkum and Kühl, 2005). Carotenoids can be classified into carotenes, which are unsaturated hydrocarbons, and xanthophylls, which present one or more functional groups containing oxygen. Beta-carotene, lutein and violaxanthin are green algae primary carotenoids, distributed within the chloroplasts along with chlorophylls. The substitution by a large variety of oxygen containing groups and their combination carries the existence of more than 600 xanthophylls (Lemoine and Schoefs, 2010). Phycobiliproteins are brilliant-coloured and water-soluble antennae-protein pigments organized in supramolecular complexes, called phycobilisomes, which are assembled on the outer surface of the thylakoid membranes (Glazer, 1994; Hemlata and Fareha, 2011). The colours of phycobiliproteins originate mainly from covalently bound prosthetic groups that are open-chain tetrapyrrole chromophores bearing A, B, C and D rings named phycobilins (Sekar and Chandramohan, 2007). Phycobiliproteins absorb energy in portions of the visible spectrum (450–650 nm) and function as accessory pigments for photosynthetic light collection (Batista *et al*., 2006). Phycobiliproteins are found in prokaryotic cyanobacteria and eukaryotic red algae (MacColl, 1998). In cyanobacteria and red algae, four main classes of phycobiliproteins are produced: allophycocyanin (APC, bluish green), phycocyanin (PC, blue), phycoerythrin (PE, purple), and phycoerythrocyanin (PEC, orange) (Bryant *et al*., 1979; Sekar and Chandramohan, 2007). In many red algae, phycoerythrins are the most abundant phycobiliproteins, while phycocyanins are abundant in cyanobacteria (Bogorad, 1975; Glazer, 1994). Different biosynthesis pathways of phycobilins have been suggested including δ-Aminolevulinic acid, heme or Biliverdin as precursors (Brown *et al*., 1984; Beale, 1993). Figure 2 shows the metabolic production of phycobilins from Biliverdin.

### Astaxanthin

#### Metabolic production and applications

Astaxanthin (3,3′-dihydroxy-β,β′-carotene-4,4′-dione) is the main carotenoid found in aquatic animals (Hussein *et al*., 2006). It serves as a precursor of vitamin A and is associated with cell reproduction and embryo development (Machmudah *et al*., 2006). Studies on food, cosmetic and medical application of astaxanthin have been undertaken because its superior antioxidant activity compared with α, β-carotene, lutein, lycopene, cantaxanthin and vitamin E. As well in other biological functions is gaining widespread popularity as human dietary supplement (Machmudah *et al*., 2006; Zhao *et al*., 2006; Yuan *et al*., 2011). The presence of this secondary carotenoid is equivalent to higher survival capacity of the cells since it enhances the cell resistance to oxidative stress generated by certain conditions of light, UV-B irradiation and nutrient conditions (Lemoine and Schoefs, 2010).

Astaxanthin can be synthetically produced or obtained from natural sources, e.g. microalgae, yeast or crustacean byproducts (Machmudah *et al*., 2006). In addition, it has unique chemical properties based on its molecular structure. The presence of the hydroxyl (OH) and keto (C = O) moieties on each ionone ring explains some of its unique features, like the ability to be esterified, to have higher antioxidant activity and more polar nature than other carotenoids. In its free form, astaxanthin is considerably unstable and particularly susceptible to oxidation (Hussein *et al*., 2006). However, free astaxathin is commercially more important than astaxanthin esters (Ma and Chen, 2001).

The green algae *Haematococcus pluvialis*, is one of the most important biological source of astaxanthin. Other microalgae species capable to accumulate secondary carotenoids are: *Botryococcus braunii, Chlamydomonas nivalis, Chlorella* sp., *Chlorococcum* sp., *Chloromonas nivalis, Coelatrella striolata* var. multistriata, *Dunaliella* sp., *Eremospherea viridis, Euglena* sp., *Neochloris wimmeri, Scenedesmus* sp., *S. obliquus, Scenedesmus komarekii, Scotiellopsis oocystiformis, Protosiphn botryoides, Tetracystis intermedim* and *Trachelomonas volvocina* (Lemoine and Schoefs, 2010). In contrast to the listed species, astaxanthin content in *H. pluvialis* represents 90% of total carotenoids. Therefore, extraction of this compound from other species represents a disadvantage in many markets due to purification steps and processing costs (Borowitzka, 2013).

**Figure 3:**
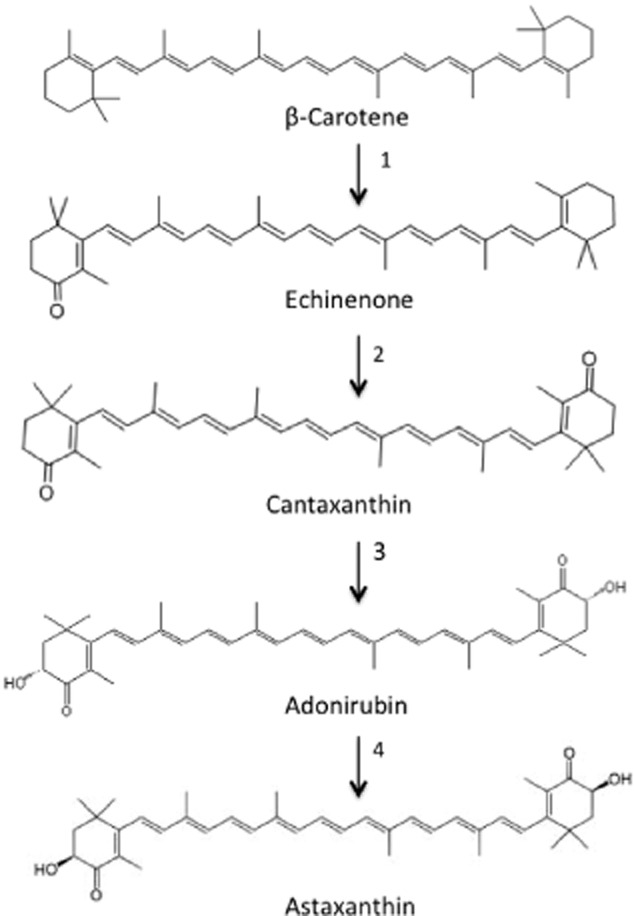
Biosynthethic pathway for the formation of astaxanthin in the microalgae *H. pluvialis* (Lemoine and Schoefs, 2010). Enzymes are 1: 4,4′- ketolase, 2: 4,4′-ketolase, 3: 3′3-Hydroxylase, 4: 3′3-Hydroxylase.

Figure 3 shows the methabolic pathway of asxtanthin production from β-Carotene in *H. pluvialis.* Astaxanthin accumulation (2–3% w/w) in *H. pluvialis* has been observed only in encysted cells. This encystment has been reported to be induced by unfavourable growth conditions such as nitrogen and phosphorus starvation, heterotrofic media, salt stress or elevated temperature (Sarada *et al*., 2002; 2006). In the absence of light, the use of suitable carbon source is crucial for attaining high biomass yield in algal culture. Moreover, nitrogen limitation in the presence of excess organic carbon substrates has increased astaxanthin production in mixotrophic cultures (Ip and Chen, 2005). The astaxanthin in encysted *H. pluvialis* cells consists of approximately 70% monoesters, 25% diesters and 5% free form (Lorenz and Cysewski, 2000).

Mature red cyst cells form a thick amorphous layer as a secondary wall inside the extracellular matrix, creating large interspace between the plasmalemma and the secondary wall (Montsant *et al*., 2001; Hejazi and Wijffels, 2004; Wang *et al*., 2004; Kang and Sim, 2007), which limits the bioavailability of astaxanthin from *H. pluvialis* (Kang and Sim, 2007).

Kobayashi and colleagues (1991) tested several carotenoids precursor in heterotrophic media. They conclude that astaxanthin production in *H. pluvialis* at 4.5 Klux for 24 h continuous lighting with acetate (14.6 mM), yeast extract 0.5 g/L, L-asparagine 2.7 mM, trace minerals and metabolites as pyruvate (12 mM), mevalonate (3 mM), malonate (3 mM) or dimethylacrylate (6 mM) were effective in carotenoid formation. Tripathi and colleagues (1999) tested various culture media for astaxanthin production in *H. pluvialis* based on Bold's Basal Medium (BBM) (Kantz and Bold, 1969) and sodium acetate, L-asparagine and yeast extract (Kobayashi *et al*., 1991). The results showed that BBM was the best for cell growth. However cells in heterotrophic medium (sodium acetate 1.99 g/L, L-Asparagine 0.4 g/L, Yeast extract 2 g/L) showed early encystment after 5 days with 1.86% w/w astaxanthin content at 1.5 Klux and 25°C in 40 days culture. Sarada and colleagues (2002) studied the influence of stress culture conditions on astaxanthin production in *H. pluvialis.* The results showed that BBM culture media at pH 7.0, salinity 0.5% (w/v) and sodium acetate (2 mM) for 12 days, allowed high astaxanthin production (1.2%w/w). Finally, further experiments based in different nitrogen sources indicated that calcium nitrate was the most effective in inducing astaxanthin formation, while sodium nitrate allowed higher biomass productivity.

Katsuda and colleagues (2004) cultivated *H. pluvialis* under Kobayashi and colleagues (1991) basal media and light emitting diodes (LEDs) at different wavelengths to study the effects of wavelength on the cells growth rate and astaxanthin accumulation. The results showed that high cell density and astaxanthin concentrations were obtained under illumination with LEDs emitting shorter wavelengths. The effectiveness of illumination at 380–470 nm for astaxanthin production was confirmed by the fact that the colour of the fermentation broths changed from green to red at 4–5 days. In addition, under illumination with the blue LEDs, the astaxanthin concentration increased to 16 μg/ml at light intensity of 8.0 μmol-photon/m^2^s while at 12.0 μmol-photon/m^2^s it increased to 25 μg/ml (astaxanthin content 6.5% of dry-cell weight). Lababpour and colleagues (2005) also reported the use of blue LEDs at 12.0 μmol-photon/m^2^s in *H. pluvialis* mixtotrophic culture to increase astaxanthin content in this algae, producing 70 μg/cm^3^ of astaxanthin in 400 h culture. Park and colleagues (2006) studied flashing light as light source for high astaxanthin production by *H. pluvialis*. The results indicated that light in short intense flashes induced astaxanthin synthesis more efficiently than giving the same amount of light energy in a continuous manner. Moreover, internally illuminated flashing light was found to be more efficient than externally illuminated flashing light. All these results indicate that light illumination induces morphological changes of *H. pluvialis*, enhancing the accumulation of astaxanthin.

Diverse authors have proposed immobilized biofilm cultivation of *H. pluvialis* in order to avoid excessive expenses of close photobioreactors (Wan *et al*., 2014; Zhang *et al*., 2014). Zhang *et al*. (2014) studied the effect of inoculum density, light intensity, nitrogen concentration and medium volume growth for astaxanthin accumulation. The optimized results showed that an inoculum density of 10 g/m^2^ and 100 μmol/m^2^s of light intensity are needed. Then, nitrogen supply (in circulating media) of 30 L of BG-11 medium (Stanier *et al*., 1971) with 1.8 mM of initial sodium nitrate strength for each square meter of cultivation surface should be supplied. Maximum astaxanthin productivity was 160 mg/m^2^d. According to the authors, this amount is higher than other productivities reported for indoor cultivation systems.

Astaxanthin is also produced by other microalgae species. Ma and Chen (2001) enhanced free *trans*-astaxanthin production on *Chlorococcum sp* from 3.6 mg/g cell DW to 5.72 mg/g cell DW when the culture was supplemented with hydrogen peroxide (H_2_O_2_) (0.1 mM) under mixotrophic (glucose 44 g/L) culture conditions at 22 μE/m^2^s for 7 days. After saponification, 7.09 mg of astaxanthin per g cell DW were achieved, with 81% as free *trans*-astaxanthin. Qin and colleagues (2008) studied the accumulation and metabolism of astaxanthin in *S. obliquus* in two-stage cultures. The results indicated that first culture stage (48 h) at 180 μmol/m^2^s and 30°C increased astaxanthin production (free and esters) with 44.6% of secondary carotenoids. In contrast, during the second stage (72 h) at 80 μmol/m^2^s and 25°C, the production of primary pigments as chlorophyll was enhanced. Ip and Chen (2005) analysed astaxanthin production of the green microalgae *Chlorella zofingiensis* in heterotrophic media with different C/N ratio with glucose concentrations (5–60 g/L) at 30°C and complete darkness. The alga exhibited the highest specific growth rate (0.031 h^−1^) and the highest growth yield (0.44 g g^−1^) at a glucose concentration of 20 g/L. However, the highest content and yield of astaxanthin were 1.01 mg/g biomass and 10.29 mg/L, respectively, at 50 g/L glucose. Therefore, glucose might be essential for providing the carbon skeleton for the formation of secondary carotenoids including astaxanthin formation in the carotenoids biosynthetic pathway. However, although nitrate is not directly involved in the biosynthetic pathway of carotenoids, it can modify the normal cellular metabolism such as protein synthesis, affecting the pigments formation in algae (Ip and Chen, 2005). In case of other nutrients, He and colleagues (2007) reported that sulfur starvation was more effective in high formation of astaxanthin compared with phosphorus or iron starvation. Therefore, culturing conditions under nitrogen and phosphate starvation, high illumination intensity, salt (NaCl) and hydrogen peroxide induce the production and accumulation of astaxanthin in algal cells.

#### Extraction and purification methods

Currently, different astaxanthin extraction methods are used in algae, including organic solvents, breakdown pretreatment process of encysted cells (cryogenic grinding and acid/base treatment), enzyme lysis (kitalase, cellulose and Abalone acetone powder mainly β-glucoronidase), mechanical disruption and spray drying (Sarada *et al*., 2006; Kang and Sim, 2007).

Since astaxanthin is a xantophyll pigment, around 95% of astaxanthin accumulated in *H. pluvialis* cells is esterified. Thus, the extraction of astaxanthin can require a hydrolisis step in order to free the astaxanthin molecule. Sarada and colleagues (2006) tested the extractability of carotenoids from *H. pluvialis* cells. Results showed that a treatment with hydrochloric acid (2N) for 10 min at 70°C followed by acetone extraction for 1 h, extracted 87% (w/w) of astaxanthin in cells without affecting the astaxanthin composition. Kang and Sim (2007) developed a two-stage solvent procedure with dodecane and methanol to extract free astaxanthin from *H. pluvialis*. The culture broth was mixed with dodecane, and the mixture was sedimented 48 h. Later, the dodecane extract was separated from the cell debris and placed in another tank and mixed with NaOH in methanol (0.02 M) at a volume ratio of 1:1 (saponification of astaxanthin esters to free form). Later, the tank was kept in darkness at 4°C (12 h) for free astaxanthin extraction in the methanol phase. The results indicated a total recovery yield of free astaxanthin of over 85% DW. Zhao and colleagues (2006) studied the effect of microwaves and ultrasound on the stability of synthetic astaxanthin isomers, concluding that microwaves induce the conversion of other astaxanthin isomers, while ultrasound degrades this pigment into colourless compounds because of cavitation produced in the solvent by the propagation of an ultrasonic waves.

Supercritical fluids (SCF) are now widely used for extraction purposes since they are more efficient than the traditional liquid solvents. Supercritical fluids act like liquid solvents with selective dissolving powers. Supercritical CO_2_ (SC-CO_2_) is by far the most common supercritical fluid used in extraction of natural compounds and food processing (Machmudah *et al*., 2006). Supercritical CO_2_ is non-flammable, non-toxic and relatively inert. Moreover, the addition of small amounts of other solvents (called entrainer or co-solvent) increase the solvent power of the SCF (Mendes *et al*., 2003). The extract obtained by SCF extraction is highly concentrated since the solvent is separated by process depressurization. Therefore, the extract is free of residues. The resulting CO_2_ gas stream can be recycled, making SC-CO_2_ extraction an environmental-friendly process (Machmudah *et al*., 2006).

Machmudah and colleagues (2006) tested different conditions to extract astaxanthin from *H. pluvialis* by SC-CO_2_. The highest amount of total extract, astaxanthin extracted and astaxanthin content in the extract were 21.8%, 77.9% and 12.3%, respectively, at 55 MPa and 343 K. Results showed that the increment in temperature did not increase extraction yields. In contrast, higher pressure increased extraction yields. This is expected since CO_2_ density increases at higher pressure, and therefore the solvent power to dissolve the substances increases. However, other compounds (lipids, proteins or polysaccharides) are also extracted leading more impurities in the extract. Total extract slightly increased at higher CO_2_ flow rate (4 ml/min) with slight increment at higher flows. In contrast, Ethanol as entrainer 1.67% (v/v) at 40 MPa, 313 K and CO_2_ flow rate of 3 ml/min increased 20% the astaxanthin extract. However, higher ethanol concentration (7.5% v/v) decreased the astaxanthin extracted, since high entrainer concentration decreases SCF density.

Fujii (2012) by SC-CO_2_ and acid treatment improved the extraction efficiency of astaxanthin in *Monoraphidium* sp. GK12 cells. The author tested also the effect of ethanol as co-solvent in the extraction yield. Results showed that at 20 MPa, 60°C for 1 h extraction time, astaxanthin yield 2.02% mg/g-dry biomass. However, the addition of 2 ml of ethanol improved the extraction yield to 2.45 mg/g-dry biomass. In case of temperature and reaction times, the author concluded that biomass/solvent ratio of 1/20 at 30°C and 15 min are sufficient for astaxanthin extraction. Finally, when the extracts were treated with acid (sulphuric and chlorhydric acid. 0.01 N in the mixture), a large portion of the chlorophyll was removed (≥ 80%).

Reyes *et al*., (2014) studied the effect that pressure, temperature and ethanol content have on the yield, astaxanthin content and antioxidant activity of *H. pluvialis* extracts during 120 min of SC-CO_2_ extraction. Also, the authors studied the effect of temperature and ethanol content using CO_2_-expanded ethanol (CXE) at 7 MPa. Optimized results showed that at 20 MPa, 55°C and 13% (w/w) ethanol content were the best conditions for SC-CO_2_ extraction, with 282.5 mg/g, 53.48 mg/g and 83% (w/w) for extraction yield, astaxanthin content and astaxanthin recovery respectively. However, with CXE extraction (50% w/w ethanol content in CO_2_ and 45°C), better results were obtained: 333.1 mg/g, 62.57 mg/g and 124.2% w/w of extraction yield, astaxanthin content and astaxanthin recovery respectively. Also, antioxidant activity in both extracts was the same. Therefore, the authors concluded that CXE extractions are much better than conventional extraction based in time difference, since just 2 h are needed for the extraction.

### Phycoerythrin

#### Metabolic production and applications

Phycoerythrin (PE) is a red coloured phycobiliprotein with absorption maxima range at 565 nm. Phycoerythrin is found in the chloroplast of cyanobacteria and red algae. In aqueous solution, phycoerythrin exists as an hexameric disk-shaped complex of (αβ)6γ subunits, with each hexamer carrying 34 bilins. There are two main classes of phycoerythrin found in red microalgae, B-phycoerythrin and R-phycoerythrin. R-phycoerythrin carries 25 phycoerythrobillins (PEBs) and nine phycourobillins (PUBs) while B-phycoerythrin carries 32 PEBs and two PUBs (Glazer, 1994).

Unlike other phycobiliproteins used as food and cosmetic colorant, phycoerythrin has advantageous physical properties that make it suitable for applications in clinical research and molecular biology. Phycoerythrin is highly fluorescent, exhibits high photostability, it has a fluorescent quantum yield independent of pH and a large Stokes shift that minimizes interferences from Rayleigh and Raman scatter (Sekar and Chandramohan, 2007; Pumas *et al*., 2012). Glazer (1994) pointed out that phycoerythrin is highly soluble in water and is negatively charged at physiological pH values, allowing minimal non-specific binding to cells, due to the fact that cells are also negatively charged. In addition, phycoerythrin can be used as label for biological molecules, as a reagent in fluorescence immunoassays, flow cytometry, fluorescence microscopy and diagnostics (Spolaore *et al*., 2006).

The microalgae species and culture conditions determine the variations in the final yield of the process. Specifically, phycoerythrin has been extracted and purified from the red algae *P. cruentum* (Tcheruov *et al*., 1993; Bermejo Román *et al*., 2002; Benavides and Rito-Palomares, 2004; Kathiresan *et al*., 2006), *Phormidium sp*. A27DM (Parmar *et al*., 2011) and the thermophile cyanobacterium *Leptolyngbya sp. KC45* (Pumas *et al*., 2012). Pumas and colleagues (2012) screened cyanobacteria species from hot spring to obtain phycoerythrin with higher temperature stability than the phycoerythrin extracted from mesophile species like *P. cruentum.* Kathiresan and colleagues (2006) studied the effect of the macronutrients present in the culture media on the production of phycoerythrin by *P. cruentum*, finding that phycoerythrin production is mainly affected by phosphate, nitrate, chloride and sulfate concentration. Other authors have determined that the synthesis of phycobiliproteins is increased under light-limiting conditions (Bermejo Román *et al*., 2002).

#### Extraction and purification methods

Phycoerythrin must be highly purified in order to meet the standards of the pharmaceutical or molecular biology field. Purity is usually determined as the absorbance ratio of A_565_/A_280_ which defines the relationship between the presence of phycoerythrin and other contaminating proteins. A purity ratio A_565_/A_280_ greater than four corresponds to diagnostics and pharmaceutical grade phycoerythrin (Benavides and Rito-Palomares, 2004). Some authors use the A_615_/A_565_ ratio to determine phycoerythrin purity in relation to phycocyanin, which is its closest contaminating protein.

Since phycoerythrin is an intracellular protein, the general purification process relies in three stages: protein extraction by cell disruption, primary recovery and purification. Disruption methods like sonication, mechanical maceration and lysozyme treatment have been successfully used to extract phycoerythrin from microalgae. Choosing the right cell disruption method has a significant impact in the recovery of the overall process. Benavides and Rito-Palomares (2006) assessed the protein efficiency release of two disruption methods, manual maceration and sonication, obtaining phycoerythrin release efficiency 5.5 times higher with sonication. Jubeau and colleagues (2012) evaluated the extraction of B-phycoerythrin from *P. cruentum* by high-pressure cell disruption varying the parameters of pressure (25–270 MPa), number of passages (1 to 3) and extraction mediums (culture media or distilled water). The authors found that contaminating proteins present in the cytoplasm are extracted at lower pressures (> 90 MPa), whereas B-phycoerythrin can be extracted at higher pressures since it is found inside the chloroplasts. Two passages achieved higher released of B-phycoerythrin, while three passages caused a loss of B-phycoerythrin due to the denaturation of the protein-pigment. Hence these authors propose a two-step selective extraction with a first passage at 50 MPa in culture medium followed by a second passage at 270 MPa in distilled water, achieving a 0.79 purity ratio.

Most authors carry out the primary recovery step by selective precipitation with ammonium sulfate. This solubility-based method is fast and fairly inexpensive. Bermejo Román and colleagues (2002) did a single precipitation step with ammonium sulfate at 65 % saturation. Parmar and colleagues (2011) recovered phycoerythrin from *Phormidium spp. A27DM* with a two-step ammonium sulfate precipitation, at 20% and 70% saturation. While Pumas and colleagues (2012) treated the cell homogenate of *Leptolyngbya sp. KC45* with ammonium sulfate at 85% saturation.

Purification is a critical step of the downstream processing of phycoerythrin. Purification is typically achieved by chromatographic methods like ion exchange chromatography, hydroxyapatite chromatography, gel filtration and expanded bed absorption chromatography. Parmar and colleagues (2011) purified phycoerythrin from *Phormidium sp*. A27DM with a single-step gel permeation chromatography using a Sephadex G-150 matrix pre-equilibrated and eluted with a 10mM trisCl buffer (pH 8.1) at a flow rate of 60 ml h^−1^. This protocol yielded a final purity ratio of 3.9. In contrast, the purity ratio achieved after ammonium sulfate precipitation was around 1.5.

Pumas and colleagues (2012) extracted and purified phycoerythrin from *Leptolyngbya sp. KC45.* Purification of the protein was carried in three chromatographic steps. The concentrated extract obtained after ammonium sulfate precipitation was treated with a hydroxyapatite column eluted with 100 mM phosphate buffer (pH 7) 0.2 M of NaCl. A yield of 37.35% and A_565_/A_280_ = 6.75 was achieved. The eluted fractions were collected and applied on a Q-sepharose column, resulting on purity ratio of 15.48 but with a low process yield of 9.65%. After a third stage performed with a Sephacryl S-200 HR resin, high purity phycoerythrin was obtained (A_565_/A_280_ = 17.3). However, extraction yield was low (1.36%). According to Kawsar and colleagues (2011) hydroxyapatite chromatography is not a reliable method since the separation ability depends on the quality of the particles, and the regeneration capacity is not good since the material might bind to other contaminating complexes.

Bermejo Román and colleagues (2002) purified phycoerythrin from *P. Cruentum* using an anionic chromatographic column of Diethylaminoethanol (DEAE) cellulose. Elution was performed as a discontinuous gradient of acetic acid-sodium acetate buffer (pH 5.5). The best results were achieved with flow rate of 100 ml h^−1^ with optimum protein loading of 7.3 mg. In addition, scale-up to a large preparative level was also tested. The capacity of the column was increased by modifying the cross-sectional area and maintaining the same superficial velocity. Final phycoerythrin recovery of 32.7% was achieved. The authors noted the importance of increasing the yield of the earlier stages since the recovery after cell disruption, and ammonium sulfate precipitation was only 45.4%.

Benavides and Rito-Palomares (2004) focused on an alternative method to concentrate and purify phycoerythrin with a polyethylene glycol (PEG)-phosphate aqueous two-phase system (ATPS) used as a single purification step. Aqueous two-phase system is an advantageous technique due to its biocompatibility and can easily be scaled. The authors found that it is possible to concentrate phycoerythrin in the PEG-rich top phase using a PEG 1450-phosphate system. The system constructed with a volume ratio (Vr) of 1, PEG 1450 of 24.9% (w/w), phosphate concentration of 12.6% (w/w) and pH value of 8 allowed the recovery of phycoerythrin with a 2.9 purity ratio. However, recovery varied depending on the parameters of the ATPS. The highest protein recovery was 73%, and the lowest recovery was 45%. Finally, purity and recovery rates were highly correlated to the system pH.

Bermejo and colleagues (2007) purified phycoerythrin from *P. Cruentum* by expanded bed adsorption chromatography (EBA) using a DEAE adsorbent. The authors focused on maximizing product recovery rather than purity, since the process is intended to replace low-resolution methods. A crude extract containing 0.033 mg phycoerythrin/ml was applied when the expanded bed reached a stable height. After processing the crude extract, the column was washed with a 50 mM acetic acid-sodium acetate buffer (pH 5.5). Finally, bound phycoerythrin was recovered by isocratic elution with a 250 mM acetic acid-sodium acetate buffer (pH 5.5). The results showed that a maximum recovery of 71% was achieved when the expanded bed volume was twice the settled bed volume (flow rate = 198 cm h^−1^), resulting an application time of 108 min. Also, the process was scaled up from a 15 mm diameter column to a 60 mm diameter column reaching a recovery of 74% with a purity ratio higher than 3. As a result, the use of EBA chromatography allowed partial concentration of the product. Therefore, EBA chromatography works as a preparative method with little product loss and is suitable to large scale production (Nui *et al*., 2010).

### Phycocyanin

#### Metabolic production and applications

Phycobiliproteins are the major photosynthetic accessory pigments in cyanobacteria and red algae. However, most studies of phycocyanin (PC) have been done in *Spirulina sp*., a cyanobacteria with high protein content. Phycobilisomes from *Spirulina sp*. consists of APC cores surrounded by c-phycocyanin (CPC) peripherally. c-Phycocyanin is the major phycobiliprotein in *Spirulina* and constitutes up to 20% of its DW. In solution, phycocyanin is found as a complex mix of monomers, trimers, hexamers and other oligomers, and its molecular weight, therefore, ranges from 44 to 260 kDa (Chaiklahan *et al*., 2012). The reported molecular mass of CPC in different cyanobacteria ranges for the oligomer between 81 and 215 kDa and that for individual subunits (α,β) between 15.2 and 24.4 kDa. In most cases, the subunits are organized in trimmers (Patel *et al*., 2005).

Phycocyanin is a colorant commonly used in food and cosmetics. In addition, it has gained importance probes for immunodiagnostics due to its fluorescence properties (Sarada *et al*., 1999). Phycocyanin is an efficient scavenger of oxygen free radicals. Therefore, therapeutic use of PC appears to be promising since many diseases are related to an excessive formation of ROS (Romay *et al*., 2003). However, the use of PC in food and other applications is limited due to its sensitivity to heat treatment, which results in precipitation and fading of the blue colour. Sodium azide and dithiothreitol are commonly used as preservatives for phycocyanin for analytical purposes, but they are toxic and thus cannot be used for food-grade phycocyanin production. In contrast, sugars and polyhydric alcohols (safe for consumption) have been used to stabilize proteins. In addition, studies have reported that modification of the protein conformation itself can improve the stability of proteins (Chaiklahan *et al*., 2012).

#### Extraction and purification methods

Allophycocyanin, a bluish green protein and CPC, a blue protein have the major absorption (*λ*_max_) in the visible region of 650–655 nm and 610–620 nm, respectively, with emission light at 660 nm and 637 nm respectively (Bryant *et al*., 1979; Sekar and Chandramohan, 2007). Determinations of these phycobiliproteins by spectrophotometry have been assessed by different authors (Furuki *et al*., 2003; Chaiklahan *et al*., 2012). The purity ratio of the phycocyanin extract is determined by the A_620_/A_280_ ratio. High purity in the extract refers to high purity ratios (Chaiklahan *et al*., 2012). Absorbance ratio ≥ 0.7 refers to food grade pigment, while reagent and analytical grade correspond to 3.9 and ≥ 4.0 respectively (Borowitzka, 2013).

Phycocyanin is water-soluble and can be easily extracted as a protein–pigment complex (Chaiklahan *et al*., 2012). General procedure includes a first extraction in buffer solutions (phosphate buffer) with sonication or ultrasound as cell disruption pretreatment. However, pretreatment times should not be long in order to avoid proteins destabilization. Later, proteins precipitation and recovery by ultracentrifugation or filtration are carried.

Sørensen and colleagues (2013) evaluated different extraction techniques for CPC extraction from the algae *Galdieria sulphuraria.* Also, all extractions were carried using PBS at pH of 7.2. The results showed contents of 25–30 mg/g of CPC for this algae. Ammonium sulfate concentration above 1.28 mol/L ensured only CPC precipitation with purity of 0.7. In case of ultrafiltration, more than 50% was lost at 100 kDa tangential flow filter, while 79% was retained by the 50 kDa filter. The authors also proposed to combine ammonium sulfate fractionation with the other methodologies tested (anion exchange chromatography, tangential flow filtration) in order to enhance purity of the recoveries (3.5–4.5).

Viskari and Colyer (1999), compared different cell disruption and extraction methods for phycobiliproteins (APC, CPC and PE) from *Synechococcus* 833. The best results were obtained using a buffer composed of 3% Chaps and 0.3% asolectin for protein solubilization combined with nitrogen cavitation for cell wall disruption. The extraction efficiencies varied between 85.2 and 87.9%.

Furuki and colleagues (2003) studied the effect of ultrasound on the extraction of phycocyanin from *Spirulina platensis*. Phycocyanin extraction was carried below 10°C, with 0.1 M phosphate buffer (pH 6.8). Later the solution was sonicated and centrifuged to remove cell debris. Results indicated that phycocyanin was not destroyed by ultrasonic irradiation as long as the irradiation time was appropriate in length. The purity of PC at 20 kHz was about 80% while at 28 kHz, the highest purity was obtained (90%).

Gantar and colleagues (2012) tested a procedure to extract, isolate and purify CPC from *Limnothrix sp.* concluding that this strain produces 18% CPC of total dry biomass. Purification of the extract was carried with chitosan (0.01 g/L) and activated carbon (1% w/v) followed of saturation with ammonium sulfate. Finally, extracts were concentrated by tangential flow filtration. The results showed that the introduction of the treatment with activated carbon and chitosan, significantly improved the purity of the pigment in the crude extract. This step increased the 620/280 ratio from 2.0 to 3.6 but also contributed to reduction of the pigment yield. Efficacy of CPC precipitation with different ammonium sulfate concentrations showed that an increment in the ammonium sulfate concentration from 20% to 25%, significantly increased the recovery of CPC. However, further increase of the ammonium sulfate concentration reduced the purity of the pigment. The final step, which included tangential flow filtration, yielded 8% CPC of dry biomass with the purity index of 4.3. In comparison, Patel and colleagues (2005) and Zhang and Chen (1999) reported 50% ammonium sulfate solution for high purity extraction of CPC in *Spirulina* since lower concentration of ammonium sulfate extracted other proteins. Therefore, Gantar and colleagues (2012) concluded that there are differences in physicochemical properties of CPC among species. In addition, the authors reported for CPC isolated from *Limnothrix* 37-2-1, an oligomeric mass of ∼50 kDa, with α and β subunits (organized in dimmers) of 13 and 11 kDa, one of the smallest molecular masses of CPC reported.

As mentioned, phycocyanin extract is not for a long term stable, since conditions as light, temperature and other microorganisms induce protein decomposition. Therefore, different studies have been assessed to increase phycocyanin's extract stability. Sarada and colleagues (1999) studied the stability of phycocyanin from *Spirulina sp.* for a period of 4 weeks at pH ranging from 2.5 to 13 using different buffers and different temperatures. The results indicated that phycocyanin was stable over a pH range of 5 to 7.5 at 25°C. However at lower temperature, phycocyanin was stable for longer periods.

Lawrenz and colleagues (2010) compared different extraction methods of phycocyanin in cell of the cryptophyte *Rhodomonas salina* and the cyanobacteria *Synechococcus bacillaris.* Also, the authors studied the effect of time storage in the degradation of phycobilins. Extractions by PBS at pH 6 were carried. Results showed that centrifugation of the crude extract (10.870 × *g*) was slightly more effective than filtration by cellulose acetate membrane. Also, for cryptophyte and cyanobacteria samples, extraction times of 4 h and 96 h, respectively, were sufficient for total pigment recovery. Finally, samples can be stored at − 80°C during 6 months without degradation.

Chaiklahan and colleagues (2012) tested the stability of phycocyanin extracted from *Spirulina sp.* by different temperature and pH with the addition of certain preservatives (glucose, sucrose, D-sorbitol, sodium chloride, ascorbic acid, citric acid, sorbic acid and sodium azide). The best results were obtained at 47°C and pH value of 6.0 with relative concentration (C_R_) value of 94%. Experiments under commercial high temperature–short time pasteurization conditions (72°C for 15 s) showed that phycocyanin remained almost intact. The C_R_ value of the solutions at pH 5.0, 6.0 and 7.0 was in 96–100% after incubation at 74°C for 1 min. In addition, phycocyanin solution was more stable at low temperature (4°C). Although the C_R_ value of the solutions at room temperature remained higher than 80% after incubation for 10 days, turbidity and odour of the solution was observed. In addition, the maximum stability at 50°C was observed at pH 6.0, while at 60°C the maximum stability was at pH 5.5. Also, glucose (20%), sucrose (20%) and sodium chloride (2.5%) were considered suitable for prolonging the stability of the phycocyanin extract. Antelo and colleagues (2008) reported that the extract of phycocyanin was longer stable at high temperature with low pH. Therefore, temperature and pH are inversely proportional with respect to the degradation of phycobiliproteins. In addition, at 50°C and 55°C, phycocyanin was more stable at pH 6.0 while at 57°C and 65°C, extract was more stable at pH 5.0. Finally, phycocyanin solution was longer stable at pH range of 5.5–6.0. In contrast, pH 5.0 leads to low stability.

## Challenges and future perspectives

Most of the world companies commercializing products from algae started producing fuels for the transport sector (biodiesel, jet fuel). However, production process is not yet economically competitive with petroleum-based fuels. Therefore, economic incentives are needed besides low price feedstocks and low price processing. Facing this situation, these companies have invested in the production of other compounds with high price commercialization, pigments and fatty acids. Currently, these products have high demand and commercialization price. However, research is needed to reduce product losses during purification steps.

As mentioned, microalgal cultivation conditions have been exhaustively explored for strains that synthesize current high-value compounds (pigments, PUFAs, polysaccharides). The extraction and purification methods for high-quality lipids, carotenoids like astaxanthin, and phycobiliproteins like phycocyanin, and phycoerythrin have been also been studied at laboratory and small-scale level. However, the recovery of intracellular metabolites at large scale is still challenging since not every cell disruption, extraction or purification methods are scalable. In addition, these technologies can be energy intensive. Nevertheless, some technologies like bead mill or high-pressure homogenization can be viable for scale-up (Munir *et al*., 2013; Ruiz-Ruiz *et al*., 2013).

In protein purification, salt removal and buffer or water exchange are key steps. In this context, membrane technologies like ultrafiltration or diafiltration commonly used in other biotechnological processes have been extrapolated to microalgae products. In the case of solvent extraction methods, the solvent has to be accepted by regulatory agencies for animal or human consumption. Solvents must also be environmentally friendly. In this context, research has been focusing on supercritical CO_2_ extraction to limit the use of other solvents like hexane, chloroform or acetone.

In the other hand, the biorefinery scheme is the key for the utilization of microalgal biomass, since high-value metabolites are viable products for researches and companies focusing on the production of biofuels. However, downstream process optimization is still a challenge faced in the large-scale production and commercialization of PUFAs and pigments. In most cases, final recoveries are low due to the number of steps required to achieve the purity levels specified by each industry. One way of reducing the number of purification steps is by process integration. Diminishing losses due to product degradation is also main concern that has to be addressed.

Currently, to increase production yields in these microorganisms, genetic modifications are being assessed. However, culture growth conditions (temperature, nutrients, light) must still be evaluated to increase the amount of specific compounds produced by the microorganisms. As well, studies in long-term stability of algae products should be evaluated. Pigments are easily degraded due to temperature, light or other microorganisms, while PUFAs oxidize by desaturation. Therefore, studies with additives or preservatives in extracts should be carried out.

Finally, economic and environmental studies regarding the production of high-value compounds from microalgae are needed. Recently, economic evaluation and life cycle analysis of some high-value compounds like phycoerythrin and astaxanthin have been carried out (Ruiz-Ruiz *et al*., 2013; Deenu *et al*., 2014; Pérez-López *et al*., 2014). In conclusion, efforts should focus in the reduction of product loss and equipment and energy costs associated with the extraction and purification steps. In addition, large-scale downstream processing must be further developed in order to achieve economically viable and environmentally friendly processes.

## Conflict of interest

None declared.
